# CT metal artifact reduction algorithms: Toward a framework for objective performance assessment

**DOI:** 10.1002/mp.14231

**Published:** 2020-06-05

**Authors:** J. Y. Vaishnav, B. Ghammraoui, M. Leifer, R. Zeng, L. Jiang, K. J. Myers

**Affiliations:** ^1^ Diagnostic X‐Ray Systems Branch Office of In Vitro Diagnostic Devices and Radiological Health, Center for Devices and Radiological Health United States Food & Drug Administration 10903 New Hampshire Ave. Silver Spring MD 20993 USA; ^2^ Division of Imaging, Diagnostics, and Software Reliability, Office of Science and Engineering Laboratories Center for Devices and Radiological Health United States Food & Drug Administration 10903 New Hampshire Ave. Silver Spring MD 20993 USA; ^3^ Canon Medical Systems, USA, Inc. 2441 Michelle Drive Tustin CA 92780 USA

**Keywords:** computed tomography, metal artifact reduction, performance evaluation, validation

## Abstract

**Purpose:**

Although several metal artifact reduction (MAR) algorithms for computed tomography (CT) scanning are commercially available, no quantitative, rigorous, and reproducible method exists for assessing their performance. The lack of assessment methods poses a challenge to regulators, consumers, and industry. We explored a phantom‐based framework for assessing an important aspect of MAR performance: how applying MAR in the presence of metal affects model observer performance at a low‐contrast detectability (LCD) task This work is, to our knowledge, the first model observer–based framework for the evaluation of MAR algorithms in the published literature.

**Methods:**

We designed a numerical head phantom with metal implants. In order to incorporate an element of randomness, the phantom included a rotatable inset with an inhomogeneous background. We generated simulated projection data for the phantom. We applied two variants of a simple MAR algorithm, sinogram inpainting, to the projection data, that we reconstructed using filtered backprojection. To assess how MAR affected observer performance, we examined the detectability of a signal at the center of a region of interest (ROI) by a channelized Hotelling observer (CHO). As a figure of merit, we used the area under the ROC curve (AUC).

**Results:**

We used simulation to test our framework on two variants of the MAR technique of sinogram inpainting. We found that our method was able to resolve the difference in two different MAR algorithms’ effect on LCD task performance, as well as the difference in task performances when MAR was applied, vs not.

**Conclusion:**

We laid out a phantom‐based framework for objective assessment of how MAR impacts low‐contrast detectability, that we tested on two MAR algorithms. Our results demonstrate the importance of testing MAR performance over a range of object and imaging parameters, since applying MAR does not always improve the quality of an image for a given diagnostic task. Our framework is an initial step toward developing a more comprehensive objective assessment method for MAR, which would require developing additional phantoms and methods specific to various clinical applications of MAR, and increasing study efficiency.

## Graphical abstract

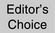

## 1 INTRODUCTION

Metal objects in computed tomography (CT) scans generate streaks known as metal artifacts. These artifacts arise from a variety of physical phenomena; mainly, beam hardening, partial volume effects, and missing projection data due to the presence of highly attenuating objects.^1^, ^2^, ^3^ Artifacts can obscure or mimic pathology, impeding detection and diagnosis of disease. Artifacts can also interfere with the radiation therapy process.^4^, ^5^

Many common implanted medical devices contain metal, including hip and knee replacements, surgical clips, dental fillings, coils, and wires. The prevalence of implanted devices is increasing; for example, between 2000 and 2010, the number of total hip replacement procedures in the United States for patients between ages 45–54 jumped 205%, from 138 700 (2000) to 310 800 (2010).^6^ In 2014, 2.5 million Americans had an artificial hip.^7^ The use of CT is increasing as well. In 1980, about 3 million CT scans per year were obtained in the United Sttaes; by 2006, that number had risen to 62 million.^8^

Concomitant with the increasing clinical significance of CT metal artifacts, an increasing number of medical device manufacturers have sought regulatory clearance from the United States Food and Drug Administration (USFDA) to market medical devices with performance claims related to the severity of metal artifacts present in CT images. Such devices have included CT metal artifact reduction (MAR) algorithms.^9^, ^10^, ^11^, ^12^, ^13^

As part of the premarket review of Class II devices with imaging indications, USFDA assesses the devices’ performance, often including artifact performance, relative to the performance of previously cleared, “predicate” devices.

In view of the large number of devices whose performance is tied to the level of metal artifact present in CT images, the lack of standardizable, reproducible methods for assessing claims related to CT artifacts poses a challenge to USFDA. Such validation methods would enable manufacturers to make claims about their device performance, and clinicians to diagnose with a better understanding of device limitations. In this work, we have explored a framework for task‐based assessment of MAR performance, which we have developed further for a single important aspect of CT MAR performance: How MAR impacts the detectability of low‐contrast lesions in the vicinity of metal implants.

### 1.1 CT metal artifact reduction — background

The first MAR techniques were based on sinogram inpainting.^14^, ^15^, ^16^ Sinogram inpainting remains the reference method for MAR, involving replacement of sinogram data corrupted by high‐attenuation areas with data linearly interpolated from nearby projections. Because the use of this method removes all information in the metal projections, it can lead to loss of contrast at metal–tissue interfaces, as well as at any edges that the removed projections crossed. Interpolation of projections also implicitly assumes that that the object has similar attenuation in all directions. When this assumption is violated — as it is in any actual clinical scenario — new artifacts can appear in the reconstructed image. In this article, we use sinogram inpainting to test our assessment framework for MAR algorithms.

Sinogram inpainting depends on segmentation of the projection data corrupted by metal. Initial segmentation can be performed on either images or sinograms; the two choices lead to two different subclasses of methods. Sinogram‐based projection interpolation^14^, ^17^, ^18^ uses the sinogram to segment the metal. Image‐domain projection completion^19^, ^20^, ^21^ first segments the metal in image space, then forward projects the segmented metal pixels to identify and remove the projections corrupted by metal, and replace them with interpolated data. While segmentation of metal in image space followed by forward projection is more involved than segmentation directly in projection space, the segmentation can be more accurate. We will use our framework to assess the performance of these two methods.

Although our article uses basic sinogram inpainting to test our assessment framework, many more recent MAR methods exist, including variants of sinogram inpainting intended to improve accuracy.^22^, ^23^ We refer the reader to Refs. ^24^, ^25^ for a review of newer methods, including iterative methods that include feedback mechanisms in their operation on image and projection data.^26^, ^27^, ^28^, ^29^

Dual‐energy CT, a technique that uses two different x‐ray spectra to image an object, also has applications in metal artifact reduction. Dual energy can generate virtual monochromatic images for specific photon energies. Generation of these virtual monochromatic images can potentially reduce metal artifacts by mitigating the beam hardening effects that arise from polychromatic x‐ray spectra. Although some studies have demonstrated the utility of dual‐energy imaging for MAR,^30^, ^31^, ^32^ others have reported that the use of dual energy in the presence of metal objects can introduce new streaks that obscure anatomical structures, or compromise the contrast to noise ratio.^33^, ^34^

Each of the major CT manufacturers markets a MAR algorithm. Other algorithms are distributed for research use only: Table 1 lists some of the MAR algorithms available in the US.

**Table 1:** Some commonly used metal artifact reduction (MAR) algorithms and their United States Food and Drug Administration (USFDA) clearance information (if available).

Manufacturer	Algorithm	USFDA 510(k) number (if available)
Canon Medical	SEMAR	K132222^10^
General Electric Healthcare	SmartMAR^35^	K163213^13^
Hitachi	HiMAR	K163528^36^
Philips Healthcare	O‐MAR	K160743^12^
Siemens Healthineers	MARIS^28^	K130901^11^
Siemens Healthineers	iMAR	K142584^9^
reVISION radiology	Metal deletion technique^37^	N/A–research use only

Although regulatory review of CT MAR algorithms is an area of ongoing USFDA effort, and this paper focuses on the assessment of MAR performance, a related area of concern is the large number of devices containing metal, with associated labeling identifying the devices as “CT compatible.” Meaningful objective assessment of MAR performance involves indirect measurement of the severity of metal artifacts, and similar methods could therefore apply to providing more precise and useful definitions of “CT compatiblity” claims. USFDA often encounters such claims in brachytherapy,^38^ which involves the introduction of radioactive sources into patients’ bodies via applicators. Treatment planning uses CT and MR scans. Since dose gradients are large in brachytherapy and slight shifts in anatomy can result in large changes in dose, applicators must be *in situ* during those scans. If the applicators generate significant artifacts, the accuracy of the reconstruction and the dose calculations have the potential to be affected. Although multiple major manufacturers market radiation therapy applicators with claims that their devices are CT compatible, no standard definition exists for this term. As a side note, radiation therapy is also a primary area in which CT MAR algorithms find application; metal artifacts may be particularly detrimental in proton therapy, where streaks on images can lead to incorrect target coverage and delineation of organs at risk.^5^

The term “CT compatible” is not limited to radiation therapy devices, appearing in the labeling of EEG electrodes, needle positioning systems, patient head positioners, and a variety of other devices: A recent Google search of “CT compatible” returned over 64 000 results, despite the fact that no standard definition of this term exists.^39^

Task‐based methods for assessment of MAR algorithms could be easily adapted to provide some objective evaluation of claims of “CT compatibility,” an application for which a regulatory need exists across different device types. Objective assessment of metal artifacts in MRI is also of interest.^40^ Although the nature of metal artifacts is different for MRI, general objective assessment frameworks developed for metal artifacts in CT could potentially find applications for other modalities as well.

### 1.2 Measuring metal artifact reduction: methods, limitations, and applications

A variety of methods have appeared in literature for measuring image quality in the presence of artifacts. These methods typically fall into one of three main categories: (a) rating scale experiments using human observers; (b) the definition and calculation from images of quantitative metrics related to artifact severity; (c) task‐based studies. In this section, we discuss the advantages and drawbacks of each method.

#### 1.2.1 Rating scale experiments using human observers

Most literature that assesses the severity of metal artifacts has relied on subjective scoring of clinical images by human observers.^15^, ^26^, ^28^, ^37^, ^41^, ^42^ The majority of such assessments have required human observers to evaluate images generated with and without MAR, and rate the images on a 5‐point Likert scale for either overall image quality or diagnostic utility. Others have used human assessments of artifact severity, with readers asked to score the most severe image artifact. In both types of experiments, the ratings yield numerical results allowing direct comparison of images with and without MAR, as well as significance testing.

An obvious advantage of human observers rating clinical images is that this setup closely resembles actual clinical practice. Rating scales distinguishing, for example, “excellent image quality with full diagnostic interpretablility” vs “good image quality allowing diagnostic interpretablility,” are also easy for patients and physicians to understand.

In addition to the expense and time involved in human studies, the disadvantage of this setup is the inherent subjectivity involved in image interpretation and assessment by humans. The subjectivity leads to inter‐ as well as intra‐observer variability: Two readers with different preferences might rank the same clinical image very differently, and even a single reader might rank the same image differently on two readings, if reader learning or fatigue are factors. In addition, human assessments are difficult to standardize or reproduce.

Additionally, in studies that generate rankings of general image quality, the image quality may not correspond directly to the suitability of an image for a given clinical task. For example, application of a MAR algorithm might reduce streaks far from an implant, yielding an image perceived as being high quality, but might reduce the detectability of lesions near a metal–tissue interface. Similarly, measurements of streak severity may not correspond directly to diagnostic utility, and also the score depends only on one of many artifacts which may be present.

Due to these limitations, USFDA believes that the use of rating scale methods is unlikely to be sufficient evidence to validate specific, and particularly quantitative, claims of artifact reduction.

#### 1.2.2 Quantitative metrics calculated from images

In lieu of rating‐scale experiments, some studies have estimated MAR performance via calculation of quantitative metrics directly from the images.

Many of these methods used phantoms with removable metal parts, enabling acquisition of images with and without metal. Such setups enable pixel‐by‐pixel comparison of the HU values^4^, ^26^, ^37^ (or quantities such as water equivalent thickness, derived from the HU values^5^) of the MAR‐corrected images or ROIs to those in the true metal‐free images. The ROI placement can focus on regions of the image that challenge the MAR algorithm.

Methods involving the pixel‐by‐pixel calculation of differences between images fall into a category of metrics called “fidelity metrics.” An advantage is their standardizability and reproducibility. A disadvantage is that image fidelity lacks a straightforward relationship to image quality for a specific task.^43^, ^44^ Fidelity‐based methods are sensitive to small changes in scale or orientation of objects, while being insensitive to tradeoffs between noise and resolution, so that two very different images can have the same fidelity.^44^ The most significant shortcoming of fidelity methods, however, is that they do not involve the actual clinical task which a reader will perform on the image. Despite having only one value of the fidelity metric, an image might be suitable for one clinical task, while being unsuitable for another.

Another quantitative method^4^ has been the use of the HU value of the brightest streak artifact as a metric. Others^45^ have counted the total number of pixels above a threshold in the MAR‐corrected images. While these approaches yield estimates of the severity of artifacts, they do not necessarily provide a measure of the overall utility of an image for a specific diagnostic task.

Still other work has defined an “artifact index”^46^ based on local standard deviation, or used measurements of overall image noise.^28^ While the noise, or changes in the noise due to application of MAR yield information about local or global image texture, they do not provide a definitive metric of image quality for a given task; for example, smoothing an image, while reducing the standard deviation of an image, can also blur the edges of a lesion.

We note that in most of the references cited above, the calculated metrics were combined with human rating scale methods, so that although both types of methods had shortcomings individually, their combination provided more meaningful information.

#### 1.2.3 Task‐based evaluation

Task‐based observer experiments are common as assessment tools in CT and other imaging fields, and can use either human or model observers. Such methods most often focus on an observer’s ability to detect, discriminate between, localize, or estimate some property of signals. Task‐based methods require defining a task representative of a clinical task, selecting an observer, and analyzing the observer’s performance on the task, that provides an objective metric of the image quality that is also relevant to clinical scenarios. We are aware of at least one study^47^ that included a task‐based evaluation of the effect of MAR on low‐contrast detectability on an arthroplasty phantom. The study used human observers. We elected to use model observers due to their comparative advantages in cost, standardizability, and reproducibility. We describe our framework in Section 2; to our knowledge, it is the first model observer–based framework for evaluation of MAR algorithms in the published literature.

The USFDA, the medical device industry, patients, and practitioners would all benefit from a standardizable method of assessing device performance with respect to metal artifacts. Task‐based assessments of image quality constitute a rigorous approach to the evaluation of imaging system performance.^48^ We have laid out and explored the feasibility of a framework that uses computer model observers to quantitatively estimate the effect of MAR algorithms on the low‐contrast detectability of lesions in the vicinity of metal implants. In particular, we designed a numerical phantom that generates simulated metal artifacts. We used a channelized Hotelling observer (CHO) to perform a lesion detection task on the numerical phantom. We demonstrated that the framework could provide an objective, reproducible, and quantitative method of assessing how MAR impacts observer performance on an LCD task.

## 2 MATERIALS AND METHODS

Our objective was to lay out a method by which we could estimate the effect of MAR on an observer’s performance at an LCD task. We also required that our framework provide the uncertainty associated with the detectability measurement, with that uncertainty being sufficiently low as to allow resolution of differences in task performance when MAR was applied, vs when it was not, in addition to differences in task performance when different MAR algorithms were applied.

Task‐based assessments of an ensemble of images require selecting a task, an observer, and a figure of merit. In this section, we provide details of these three components of our assessment.

### 2.1 Task

A principal clinical task related to MAR is detection of lesions in the presence of hip implants, dental fillings, cochlear implants, deep brain stimulators, and other objects containing metal. In trauma situations, including battlefields, metal artifacts also arise from bullets or shrapnel.^49^, ^50^ We focused on assessing how MAR affects an observer’s performance at detecting low‐contrast lesions in the presence of metal artifacts.

Signal detection is one of only several clinically relevant tasks for which an observer’s performance may be impacted by the application of MAR. Other clinical tasks performed in the presence of MAR include segmentation of organs in radiation therapy applications, and assessment of metal‐bone interfaces in orthopedics. Ideally, a framework for assessing MAR performance for a specific clinical task should use a study assessing the effect of MAR on an observer’s performance at the appropriate type of task.

We designed a digital reference object; a numerical phantom that resembled a human head. The anthropomorphic aspects of the phantom are somewhat incidental; the phantom was not intended to be directly representative of a specific clinical situation, but rather to incorporate strongly attenuating metal in some geometries that loosely mimic some commonly found clinical ones — for example, oral lesions can be obscured by metal artifacts resulting from dental fillings.

**Figure 1:**
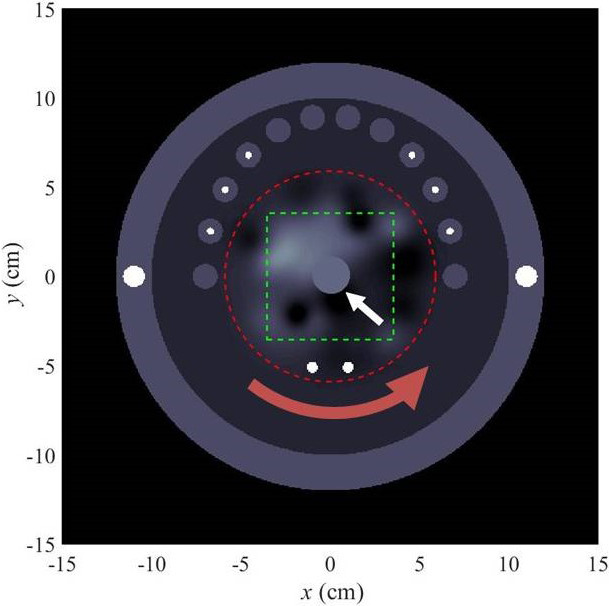
Schematic of phantom. The phantom consists of an sk, an inner disk, and a rotatable inset (interior of the red dashed circle) with an inhomogeneous, lumpy background. The curved red arrow indicates that the inset rotates. The phantom contains metal in locations that are motivated by dental fillings, cochlear implants, and deep brain stimulators. At the phantom center (white arrow) is a low‐contrast signal. This signal corresponds to an interchangeable rod, so it replaces the lumpy background rather than being added to it. Rotating the inset to a different, random angle at each acquisition randomizes the background as well as the relative position of artifacts, avoiding overtraining the observer on a particular phantom configuration. All metal objects outside the inset are at fixed positions. Signal detection studies use the region of interest indicated by the green dashed square. [Color figure can be viewed at wileyonlinelibrary.com]

The phantom (schematic in Fig. 1) includes teeth with metal fillings, as well as two disks (corresponding to metal rods on a physical phantom) in the positions of cochlear implants, and two in the position of deep brain stimulators. In the middle of this phantom is a rotatable inset. The signal, when present, is a disk located at the phantom center. On a physical phantom, this signal would be a removable rod. We selected a range of signal contrasts that permitted us to examine the full range of observer performance; the lowest contrast we examined was nearly undetectable by the observer; the highest contrast was easily detectable. Although we did not examine negative contrast signals, negative contrasts can also be clinically relevant in many situations; our framework does not change for negative contrast signals.

The inset has inhomogeneous attenuation, generating a lumpy background on the image. Various methods exist for generating such inhomogeneity in a real phantom: An example of a phantom capable of generating multiple realizations of a random, textured background for use in observer studies occurs in the $M^{3}R$ framework.^51^ In this case, 3D printing could generate a textured inset that could be rotated between acquisitions. Alternately, the inclusion of movable objects within the phantom could make the phantom dynamic, yielding a different image with each acquisition.

For each material in the phantom, we adapted publicly available software^52^ to interpolate values of the energy‐dependent attenuation *μ*(*E*) from data in the NIST XCOM photon cross section library.^53^ For convenience, this phantom consists of geometric primitives: disks and Gaussians centered at various radii. The Radon transform of each of these objects is analytic — a useful feature in numerical simulation of the projection data. Table 2 describes the geometric primitives that make up the phantom.

**Table 2:** Phantom parameters. The center of the phantom corresponds to (0 cm, 0 cm).

Disk	Coordinates of center (cm)	Radius (cm)	Attenuation
Outer disk	(0,0)	12	Bone
Inner disk	(0,0)	10	Water
Inset	(0,0)	5.9	1.05 × water
Teeth	(7 cos (*nπ*/11),9 sin (*nπ*/11)), *n* = 0,1,…,11	0.7	Iron
Cochlear implants	(±11, 0)	0.6	Iron
Deep brain implants	(±1, −5.1), rotate with inset	0.3	Iron
Dental fillings	Same as teeth	0.2	Iron
Signal (when present)	(0,0)	0.5	(1.00–1.06) × background

We experimented with various different metals, and also with removing projection data directly rather than simulating metal. Ultimately to balance realism with simplicity and also create artifacts strong enough to challenge the MAR algorithms, we selected the strongest attenuator (iron) for all metal in the phantom.

The attenuation of the lumpy background is a sum of *N* Gaussians,

$$\sum_{i=1}^{N}\mu_{i}e^{-\pi |r-r_{i}{|}^{2}/w_{i}^{2}}$$

where **r** = (*x*,*y*), $r_{i}$ , $w_{i}$ , and $\mu_{i}$ determine the centers, widths, and amplitudes of each Gaussian, respectively. We have selected *N* = 20, $w_{i}=1.3\;+\;2R$ with *R*∼*N*(0,1) a normally distributed random variable, and $r_{i}$ also random, with the constraint that $r_{i}<4.9$ , to keep the lumps confined within the inset radius. The lumps rotate with the inset. The amplitude of each lump is $\mu_{i}\;=\;\mu_{\mathrm{water}}\times 1.5R$ , where *R*∼*N*(−1,1).

Using this phantom, we generated projection data that we reconstructed with FBP only, MAR 1, and MAR 2, respectively. The task that an observer performed was determining whether a signal was present at the center of the phantom. We generated each image with the inset oriented at a random angle, corresponding to the rotation of the phantom inset between acquisitions. We introduce this random component in order to prevent overtraining the model observer on a particular pattern of streak artifacts.

The phantom contains a removable disk signal at the center. The signal can be either present (in which case it is a homogeneous disk at the center) or absent (in which case the surrounding lumpy background is present at the center). At each value of imaging parameters examined, we generated 1000 512 × 512 images, with the inset at a different, random angle for each acquisition. From each image, we extracted a 121 × 121 pixel ROI centered on the origin, with the signal at the ROI center. (To make the study more efficient, when using a physical phantom, it would also be possible to make use of ROIs at other locations or values of *z*).

### 2.2 Simulating metal artifacts

Having designed the phantom in Fig. 1, the next task is to simulate x‐ray projection data using the phantom. Metal artifacts in CT arise from a variety of physical phenomena^1^, ^2^ whose net effect is to corrupt the projection data, resulting in the breakdown of a linear relationship between the log‐attenuation and the measured projections. Since the goal of our study was to demonstrate the feasibility of using our framework of MAR evaluation, we used the simplest possible simulation containing the basic physics of metal artifact generation.

We generated noisy projection data^2^, ^54^, ^55^ including constant scatter, Poisson noise, and normally distributed electronic readout noise. We generated a tube spectrum from Siemens’ online x‐ray spectrum–generating tool^56^ for a tube voltage of 120 kVp. To generate attenuation maps of the phantom, for each material present, we adapted publicly available software^52^ to interpolate values of the energy‐dependent attenuation *μ*(*E*) from data in the NIST XCOM photon cross section library.^53^ We reconstructed the resulting sinograms using filtered backprojection (FBP) and a Ram‐Lak filter as implemented in matlab.^57^ Note that because the phantom consists entirely of geometric primitives (disks and Gaussians) whose Radon transforms are analytic, fully analytic reconstruction is possible.

### 2.3 Metal artifact reduction methods

The MAR algorithms we implemented were two simple variants of sinogram inpainting, described in Section I. We denote these methods MAR 1 and MAR 2. In MAR 1, we segmented the metal from the sinogram. In MAR 2, we segmented the reconstructed image to identify the metal, then forward projected the metal pixels to identify the corresponding data on the sinogram. The metal pixels on the sinogram were then replaced by values interpolated from the remaining data.

As our MAR algorithms were rudimentary, we selected the threshold for metal segmentation manually, for parameters (dose, signal amplitude) in the middle of our parameter space. These thresholds were not set to automatically adapt to changing parameters. Segmentation algorithms can be sensitive to noise as well as to absolute CT number. With changes in dose, we anticipated that MAR 1 might fail completely, providing the opportunity to check whether our assessment method was able to correctly identify this failure.

### 2.4 Observer

We reviewed the use of model observers in CT image quality assessment in Ref. 58. For an overview of channelized model observers, we refer the reader to Ref. 48. To summarize briefly, channelized observers perform feature extraction on images, reducing high‐dimensional image data to a few relevant features. Each channel is an image corresponding to a feature, and the channel output is the scalar product of that channel with the image.

To determine signal detectability, we applied a channelized Hotelling observer (CHO), described on pp. 936–937 of Ref. 48. The CHO is designed for binary discrimination tasks such as, in this case, detection of a lesion at the center of the phantom. The goal is to classify each image as belonging to class 1 (lesion absent) or class 2 (lesion present). The signal is known exactly, and the background, while known exactly, is both spatially inhomogeneous and rotated to a random angle for each acquisition (see Fig. 1).

We refer the reader to^48^, ^58^ for information about different options for channels. While USFDA does not specify the particular model observer or types of channels that manufacturers should use in demonstrations of imaging CT performance, the observer and channels should be motivated by human performance. In this case we have elected to use ten dense difference of Gaussian (DDOG) channels; DDOG channels have been validated against human performance, with which they correlate well, following noise regularization.^59^ We determined the number of channels by investigating AUC as a function of channel number. We found that the task in this situation was a low‐order one, for which the AUC was above 0.5 only for the first seven channels, so that the use of ten channels was sufficient. In Section 2.E we discuss the AUC as a measure of observer performance.

### 2.5 Figure of merit

The AUC is a common summary figure of merit for observer performance. Here, we describe the estimation of the AUC. After extracting ROIs from each image, as shown in Fig. 1, the ROI pixel values were cast as a *q*,× 1 column vector, **g**. Define *p*≪*q* as the number of channels. The weights defining each channel were collected into respective columns of a *q* × *p* channel matrix, **U**, which upon application to each image yielded a *p* × 1 channel output vector $v\;=\;U^{T}g$ . In our study, $q\;=\;121^{2}\;=\;14\;641$ and *p* = 10.

Following the notation and methods of Wunderlich and Noo,^60^ we denote the means and covariance matrices of $v_{i}$ (*i* = 1,2) as $\mu_{i}$ and $\Sigma_{i}$ , respectively. Define $\Delta \mu \;=\;\mu_{2}-\mu_{1}.$ In addition, let $\bar{\Sigma }\;=\;\left(\Sigma_{1}+\Sigma_{2}\right)/2$ . In this notation, the template for a CHO is $w\;=\;{\bar{\Sigma }}^{-1}\Delta \mu$ . We assumed that $\Sigma_{1}\;=\;\Sigma_{2}\;=\;\Sigma$ , simplifying the CHO template to

(1) $$w=\Sigma^{-1}\Delta \mu .$$

To classify an image with a channel output vector **v**, the CHO starts by generating a test statistic, $t\;=\;w^{T}v$ . We assume that the test statistic *t*, is normally distributed for each class. In the presence of strong artifacts, the assumption that *t* remains normal requires some justification. The majority of the streak artifacts are nonrandom in our experiment, as they arise from implants with fixed locations relative to the signal and background. These artifacts contribute to the image at the signal location in a deterministic way. As a result, the randomness of the test statistic is primarily influenced by the statistical variations in the pixel values due to the photon counting noise at the detector, which is transferred to the image domain during the reconstruction process. There is also a contribution to the randomness in the image values at the signal location from the variation in streaks due to the random rotation of the two high‐contrast implants

The test statistic *t* is a weighted sum of image values in the area of the expected signal. As per the Central Limit Theorem, the sum of a large number of random variables is asymptotically Gaussian, even in the case of as few as ten random values, and regardless of the probability distribution functions of those variables.

The observer signal to noise ratio (SNR), a figure of merit related to the AUC, is then

(2) $${\text{SNR}}^{2}=\frac{|{\bar{t}}_{1}-{\bar{t}}_{2}{|}^{2}}{\frac{1}{2}\left(\sigma_{t_{1}}^{2}+\sigma_{t_{2}}^{2}\right)}$$

with the AUC then being

(3) $$\text{AUC}=\Phi (\text{SNR}/\sqrt{2}),$$

where Φ(*x*) is the cumulative distribution function for the standard normal distribution (see p. 819 of Ref. 48).

In practice, estimates of the AUC use a finite set of images. The finiteness of the image set can introduce bias in the estimate.^61^, ^62^, ^63^ We now consider two methods of estimating the AUC that provide upper and lower bounds on the bias: The resubstitution and hold‐out methods. In general, resubstitution estimates of the AUC are positively biased, and are higher than the AUC for an infinitely trained CHO, whereas estimates obtained via hold‐out are lower.^61^ Using both methods, and identifying the value of AUC to which they converge as the number of images becomes infinite, can provide an unbiased estimate of the AUC.^61^

The first method we examine is the resubstitution method, which uses the same $N_{T}$ images for training and testing the observer. Resubstitution uses the full $N_{T}$ images to compute all terms in Eq. (2), which yields the AUC via Eq. (3). While resubstitution has the advantage of simplicity and efficiency, because one does not need to partition the data set, the result is positively biased due to re‐use of the data and the finiteness of the image set.^64^

The second strategy, called the hold‐out method, uses independent image sets for training and testing, with the training set containing $N_{T}$ images and the test set containing the remaining $N-N_{T}$ images, where *N* is the total size of the image set.

Estimating the AUC using the hold‐out method requires calculating different terms of the SNR in Eq. (2) using either the training set or the test set. Training the observer, that is, calculating the Hotelling template in Eq. (1) uses only the training images. Testing the observer means applying this template to the images in the test set, that is, calculating the test statistics $t_{i}\;=\;w^{T}v_{i},\left(i\;=\;1,2\right)$ with the $v_{i}$ computed using only the test images. Eqs. (2) and (3) then yield the AUC.

**Figure 2:**
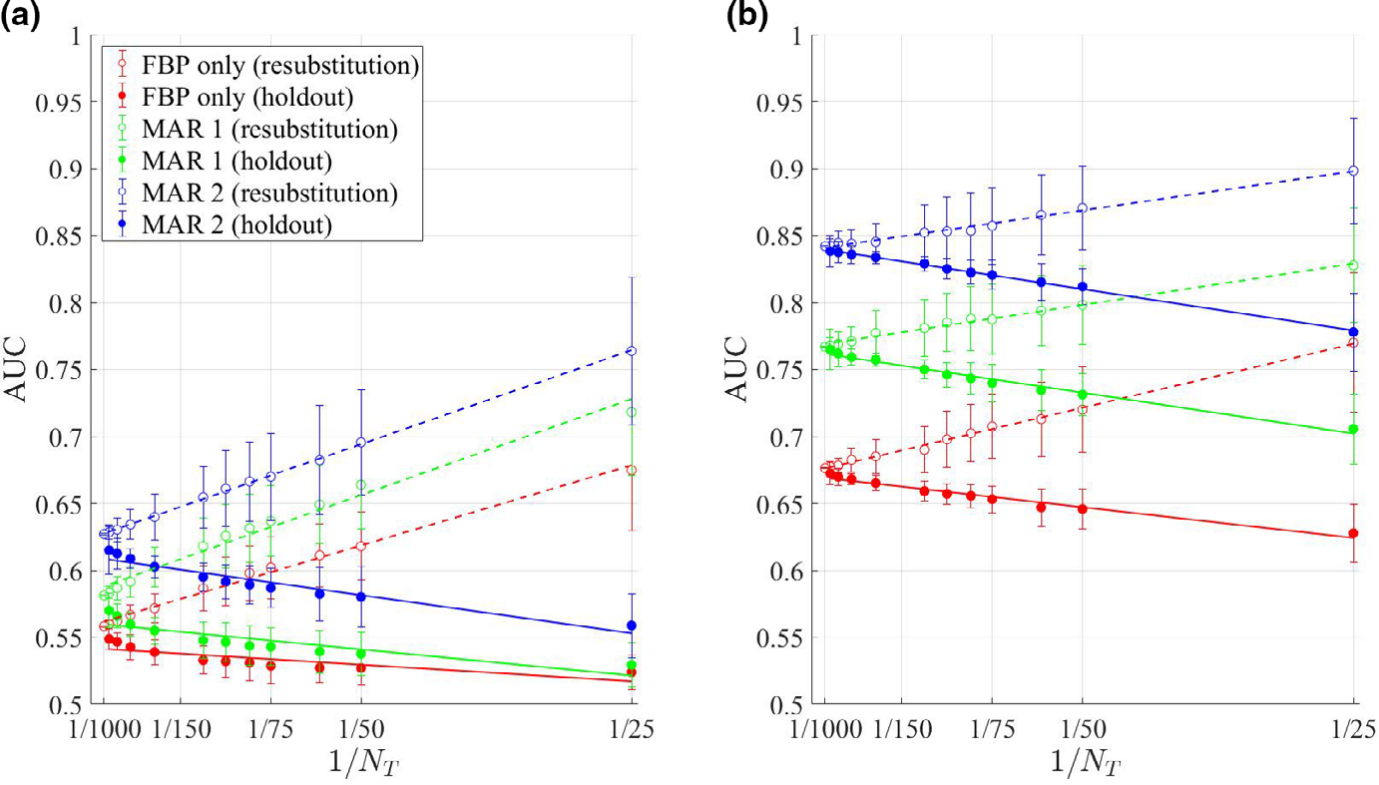
These plots compare observer performance at a signal detection task with no metal artifact reduction (MAR) (red), MAR 1 (green), and MAR 2 (black). The two plots in (a) and (b) are “antler plots”^61^, ^62^ demonstrating variation of area under the ROC curve (AUC) with $1/N_{T}$ , the size of the training set (using the hold‐out method) or the size of the image set (using resubstitution). The signal amplitudes are 1.01 (a) and 1.03 (b) times background. We include both figures in order to demonstrate that as the separation between AUC values changes, one might need different numbers of images to establish the existence of a statistically significant difference in performance. The error bars shown are the standard deviation of the values over 50 repetitions of the measurement. [Color figure can be viewed at wileyonlinelibrary.com]

The resubstitution and hold‐out results together generate an “antler plot” (Fig. 2), a version of the “learning curve” used to examine the performance of machine learning classifiers.^63^ The antler plot shows the AUC estimated via the resubstitution and hold‐out methods as $N_{T}$ varies from 25 to 1000. At each value of signal contrast, we averaged AUC results over 50 different image sets, selected randomly; the error bars indicate the standard deviation over these 50 measurements. As expected,^61^, ^62^ the AUC obtained by both methods has power‐law dependence on $N_{T}$ . The resubstitution method demonstrates positive bias, whereas the hold‐out method is slightly negatively biased. Denote the value of the AUC to which the resubstitution and holdout methods converge as $N_{t}\to \infty$ as ${\text{AUC}}_{\infty }$ — this would be the unbiased AUC calculated from an infinite number of images. We can obtain an estimate of ${\text{AUC}}_{\infty }$ by fitting the antler plot data to lines; ${\text{AUC}}_{\infty }$ is the *y*‐intercept of both lines, at which the resubstitution and hold‐out methods converge as the training set becomes large.

Note that the appropriate size of $N_{T}$ depends on the specific claims that an investigator wishes to make. For example, resolving the large difference between MAR 2 and FBP performance is easier than resolving the finer difference between MAR 2 and MAR 1 performance. Establishing any performance differences is more difficult at lower signal contrast [Fig. 2(a)] than at higher signal contrast [Fig. 2(b)], where the performances are better separated.

To gain an overall understanding of performance, generating antler plots for a few different values of signal amplitude is helpful. For example, in Fig. 2(b), resubstitution with 100 images is enough to resolve differences. In Fig. 2(a), closer to 500 images would be required.

## 3 RESULTS

**Figure 3:**
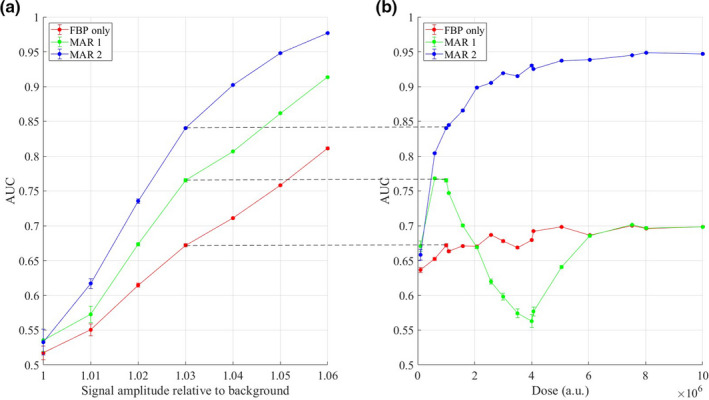
Detectability curves, demonstrating variation of area under the ROC curve with (a) the amplitude of the disk signal relative to the background, dose fixed at 1e6 a.u., and (b) dose, signal amplitude fixed at 1.03 times background. Dashed lines indicate equivalent points. The data in green demonstrate the nonlinearity of metal artifact reduction algorithms, in terms of performance vs dose. [Color figure can be viewed at wileyonlinelibrary.com]

Using the antler plot formalism and methods in Section 2, we generated detectability curves (Fig. 3). These curves are the final product of our framework for MAR assessment, summarizing the performance of our CHO on two different MAR algorithms as well as FBP alone, over a range of signal amplitudes [Fig. 3(a)] and radiation doses [Fig. 3(b)] . The error bars are smaller than the markers.

Each point on the curves was calculated by extrapolating the resubstitution and holdout arms of the antler plot to $1/N_{T}\;=\;0$ and calculating the intercepts, denoted as ${\text{AUC}}_{\infty }^{\left(r,h\right)}$ . Each measurement was repeated 50 times using random image sets. ${\text{AUC}}_{\infty }$ was then estimated as ( $\left\langle {\text{AUC}}_{\infty }^{\left(r\right)}\right\rangle +\left\langle {\text{AUC}}_{\infty }^{\left(h\right)}\right\rangle )/2$ , where the angle brackets denote the mean over the 50 repetitions.

To estimate the error, we calculated contributions from bias and variance. The maximum error due to bias was estimated as one half the difference between the values of ${\text{AUC}}_{\infty }$ obtained by the resubstitution and holdout methods: $\epsilon_{b}\;=\;\left|{\text{AUC}}_{\infty }^{\left(r\right)}-{\text{AUC}}_{\infty }^{\left(h\right)}\right|/2$ . The error due to variance $\epsilon_{v}$ was estimated using the standard deviation of the mean of ${\text{AUC}}_{\infty }$ over 50 repetitions. The root‐mean‐square error *ε* was calculated as $\epsilon \;=\;\sqrt{\epsilon_{b}^{2}+\epsilon_{v}^{2}}$ , to include contributions from bias and variance. The errors are of the order $10^{-3}$ and are too small to be visible on Fig. 3.

Note that, although sufficient for this framework, error bars calculated in this manner underestimate the true error, as the samples used to calculate the standard deviation are actually correlated. A more thorough error analysis would make use of a multireader, multicase variance analysis involving model observers trained on separate image sets, and tested on an independent set of images. The USFDA has published a software tool to assist with the setup of such analyses.^65^

The curves in Fig. 3(a) show the AUC as a function of signal amplitude. The results are as expected. At all values of the signal amplitude, both MAR algorithms improve the detectability of signals. At all values of signal amplitude, MAR 2 (image‐based metal segmentation) outperforms MAR 1 (sinogram‐based metal segmentation), with statistically significant differences in the results.

**Figure 4:**
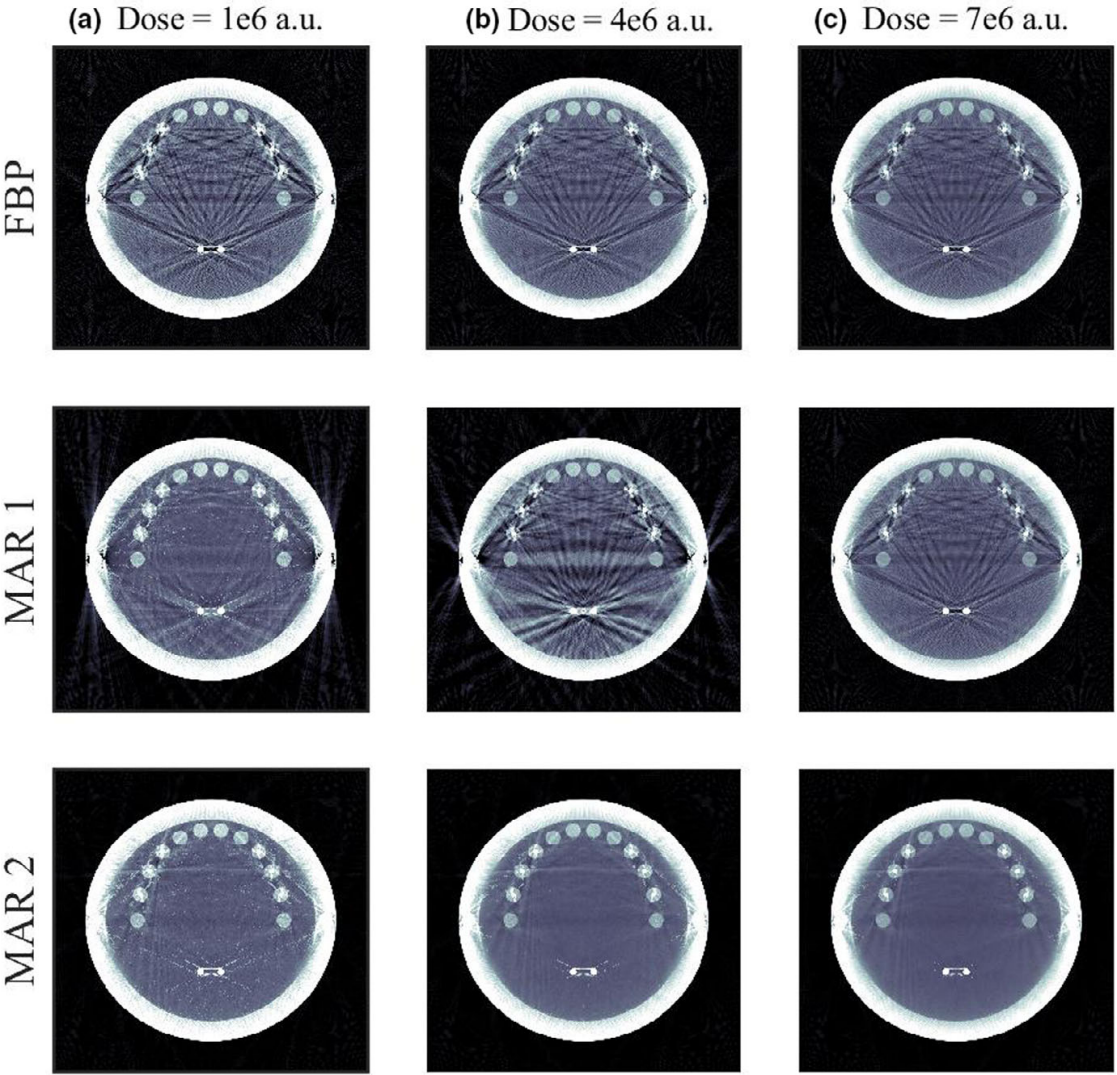
This figure illustrates the detectability data laid out in Fig. 2. These images show the performance of filtered backprojection, metal artifact reduction 1 (MAR 1), and MAR 2 at three different doses (a–c). MAR 1 involves sinogram‐based thresholding; the procedure is more robust to changing signal amplitude, but is fairly sensitive to changes in image noise. At intermediate doses, MAR 1 begins to introduce artifacts, before failing completely in Panel (c). MAR 2 performance, in contrast, increases as the dose goes up. The detectability curves identify these features. [Color figure can be viewed at wileyonlinelibrary.com]

However, when we vary the dose (right), we see that the above observation does not hold for all doses. For a range of doses, the application of MAR 1 in fact degrades the image as compared to FBP. The failure of MAR 1 occurs because MAR 1 attempts to segment metal projections in sinogram space, a process that is very sensitive to image noise. A threshold that is satisfactory at one level of dose quickly breaks down at other doses until at different noise levels, the thresholding is unable to identify metal pixels, and MAR 1 has no effect on the image at all. By looking at the images corrected by MAR 1 (Fig. 4), the breakdown is visually obvious — and our performance assessment framework is able to identify it.

An unusual feature of this setup is that we do not expect an AUC equal to 0.5 when the signal amplitude is equal to the background, that is on the left hand side of the plots in Figs. 3(a)–3(b). The lumps are Gaussian with random widths, whereas the signal is a homogeneous disk. Even when the signal amplitude is equal to the mean background, a small but discriminable difference exists between the signal present and signal absent case, and the observer is capable of identifying this difference.

## 4 DISCUSSION

We have explored a potential framework for assessing MAR algorithm performance by developing phantoms for clinical tasks that are potentially affected by MAR, examining how MAR affects a model observer’s task performance, and statistically analyzing the results. In particular, we have explored the feasibility of this framework for LCD tasks, by using computer model observers to examine the effect of two sinogram inpainting algorithms on model observer performance. Our results showed that the observer’s task performance in the presence of MAR was strongly dependent on object and imaging parameters, suggesting that MAR algorithm validation should cover the full range parameters over which algorithm performance is indicated. Our model observer‐based framework for assessing MAR performance was able to successfully detect the failure of the MAR algorithms as we changed parameters.

Data obtained via model observer frameworks may be useful as partial support for labeling claims on MAR performance. For example, for MAR 2, the data in Fig. 3(b) might support a statement like “In a simulation study based on a numerical head phantom with metal implants, MAR 2 improved low‐contrast signal detectability over a range of signal amplitudes and doses as compared to both MAR 1 and FBP alone.”

The data in Fig. 3(b), which show a maximum AUC difference of approximately 0.2 between FBP and MAR 2 might also support the addition of more quantitative statements such as “MAR 2 improved the AUC by up to 0.2 over FBP alone.” (A quantitative comparison to MAR 1 might be misleading, since over a range of doses, MAR 1 did not perform as well as FBP.)

The quantitative results were consistent with the results we would have expected based on visual assessment of the images. To more completely characterize the effect of a MAR algorithm on signal detectability, we recommend that investigators present the full performance curves as functions of object and imaging parameters, such as signal amplitude and dose. As illustrated by the results in Fig. 3, presenting the entire detectability curve provides a more complete understanding of each MAR algorithm’s performance characteristics, as well as an understanding of the particular parameters at which any comparison is made. A summary statistic for the difference in algorithm performance based on the difference in the areas under these curves might be a useful alternative to point‐based measures; however, the development of such a statistic would require further investigation. Another important point is that MAR algorithms may work well far from metal–tissue interfaces, while obscuring details in an image that are close to metal objects. A methodology such as the one described in this paper might allow detailed exploration of those limitations, and development of either appropriate performance claims or disclaimers.

Although the main forms of CHO channels have been extensively validated against human performance, little work has been done in the area of validating CHO channels in situations where the image has strongly oriented features (streaks). Further investigation along these lines would be valuable.

We would envision that an investigator intending to make claims regarding MAR performance would be responsible for validating the results of simulation studies with a limited dataset using actual images of a physical phantom, to demonstrate that the simulation is representative of actual device performance. Clinical images could also be a useful supplement.

At present, our framework is simulation based and therefore the need for many images is not a problem. Practically, however, CT scanners are not designed to allow repeated scans of a phantom in a short period. To make our framework practical using real data, we would want to obtain statistically significant results using tens of images. Many options exist to optimize the framework we describe in this paper to be less burdensome. For example, if the phantom is designed with multiple signals in the inset, multiple ROIs could be obtained from a single acquisition. Careful consideration would have to be devoted to the size of the ROIs vs the size of the signals, as well as the selection of ROIs. ROIs could also be extracted at different *z* positions.

In the context of validation of radiation dose reduction by iterative reconstruction algorithms, Popescu and Myers^66^ have demonstrated that the use of signal localization tasks is potentially much more efficient than the use of signal detection tasks.

The application of iterative reconstruction algorithms can sometimes result in metal artifact reduction, despite the algorithms not having been designed with that specific purpose in mind.^42^ The fundamentals of our framework for assessing MAR are independent of the nature of the MAR algorithm; a similar framework could be applied to assess whether iterative reconstruction can enhance low‐contrast detectability in the vicinity of metal, as compared to FBP.

Although we have designed our methods with multidetector CT in mind, it is likely that the general framework can be adapted for use in dental cone beam CT, particularly since we used a head phantom.

We note that while some clinical tasks that MAR potentially impacts involve detection of low‐contrast lesions — for example, detection of malignant rectal lymph nodes in the presence of hip implants; oral lesions in the presence of artifacts from dental fillings — others do not. In the context of radiation therapy, for example, MAR is applied to segmenting organs — a task that depends on the precise delineation of the organ boundaries, as opposed to LCD. In some orthopedic applications, accurate reconstruction of the bone–metal interface is a priority. A complete task‐based framework for MAR evaluation would include multiple phantoms and tasks reflecting multiple clinical scenarios, as well as analysis methods.

The USFDA envisions public and validated tools supporting the types of analyses described in this paper. These tools would include digital phantoms, models of the imaging physics, and methods for analyzing the resulting images and generating metrics of image quality. Some of these tools, especially the statistical analysis tools, are amenable to standardization, while others, like the phantom, might require customization to reflect a particular vendor’s hardware or MAR algorithm. Although manufacturers’ individual MAR algorithms are proprietary, phantoms, models of the observer, and statistical analysis of the results can exist outside of the competitive space, so that the potential exists for collaboration on shared development, dissemination, and validation of better phantoms and accurate simulation tools. Collaborations of this type could potentially lead to increased reliance on simulation tools for system evaluation and future regulatory decision‐making. The development of such simulation tools is a priority to USFDA.^67^, ^68^, ^69^

The utility of image quality assessment in the presence of metal artifacts extends beyond the evaluation of MAR algorithm performance, and has the potential to provide more precise information about artifact generation than the plethora of “CT compatibility” claims presently made regarding medical devices containing metal. Such assessments would be valuable to vendors and to consumers of the devices.

## 5 CONCLUSIONS

We explored the feasibility of a potential task‐based framework for the objective assessment of the effect of CT MAR algorithms on low‐contrast detectability, which is an important component of overall MAR performance. Other phantoms, tasks, and analysis methods could be developed to assess other aspects of MAR performance.

We tested our framework on two sinogram inpainting algorithms: Sinogram‐based and image‐based projection completion. Our framework involved the use of a signal detection task in the presence of a variable background, the application of a model observer validated against human performance, and ROC‐based methods for statistical analysis of the results.

We demonstrated that our method was able to distinguish observer performance in the presence of MAR from that when MAR was not used. Furthermore, the method was able to distinguish observer performance in the presence of two different MAR algorithms. We also found that MAR algorithms do not always enhance the detectability of low‐contrast lesions in the presence of metal. Rather, the degree to which they improved this task‐specific aspect of image quality depended sensitively on parameters like signal amplitude and dose, and that if applied in incorrect parameter regimes, MAR could in fact degrade lesion detectability.

The USFDA encourages the use of simulation as partial support for claims regarding artifact reduction. Simulation offers the advantage that large numbers of images, and new, complex objects are available. However, in some cases, simulation results may need to be validated with a dataset based on actual phantom images; additional work is required to optimize the framework in this paper to be suitable for use with practical numbers of real phantom images. Many options exist for this optimization, including the use of search tasks, which can potentially yield the same statistical power using fewer images.^66^

## CONFLICT OF INTEREST

This work was performed when JYV was employed at the USFDA. The authors have no relevant conflict of interest to disclose.

## References

[1] Bushberg JT , Seibert JA , Leidholdt EM Jr., Boone JM . The Essential Physics of Medical Imaging, 2nd edition. Philadelphia: Lippincott Williams & Wilkins; 2002.

[2] De Man B , Nuyts J , Dupont P , Marchal G , Suetens P. Metal streak artifacts in X‐ray computed tomography: a simulation study. IEEE Trans Nucl Sci. 1999;46:691–696.

[3] De Man B , Nuyts J , Dupont P , Marchal G , Suetens P. Reduction of metal streak artifacts in x‐ray computed tomography using a transmission maximum a posteriori algorithm. IEEE Trans Nucl Sci. 2000;47:977–981.

[4] Huang JY , Kerns JR , Nute JL , et al. An evaluation of three commercially available metal artifact reduction methods for CT imaging. Phys Med Biol. 2015;60:1047. 25585685 10.1088/0031-9155/60/3/1047 PMC4311882

[5] Andersson KM , Dahlgren CV , Reizenstein J , Cao Y , Ahnesjò A. Thunberg P. Evaluation of two commercial CT metal artifact reduction algorithms for use in proton radiotherapy treatment planning in the head and neck area. Med Phys. 2018;45:4329–4344. 30076784 10.1002/mp.13115

[6] Wolford M , Palso K , Bercovitz A . Hospitalization for total hip replacement among inpatients aged 45 and over: United States, 2000‐2010. NCHS data brief, no 186, Technical report,National Center for Health Statistics, Hyattsville MD; 2015. 25714040

[7] Kremers HM , Larson DR , Crowson CS , et al. Prevalence of total hip and knee replacement in the United States. J Bone Joint Surg. 2015;97:1386. 26333733 10.2106/JBJS.N.01141 PMC4551172

[8] Brenner DJ , Hall EJ. Computed tomography ‐ an increasing source of radiation exposure. N Engl J Med. 2007;357:2277–2284. 18046031 10.1056/NEJMra072149

[9] Siemens Medical Systems, Inc. 510(k) Summary for iMAR. https://www.accessdata.fda.gov/scripts/cdrh/cfdocs/cfpmn/pmn.cfm?ID=K142584; 2015.

[10] Toshiba Medical Systems Corporation . 510(k) Summary for the Aquilion ONE Vision, V6.0. https://www.accessdata.fda.gov/scripts/cdrh/cfdocs/cfpmn/pmn.cfm?ID=K132222; 2013.

[11] Siemens Medical Systems, Inc . 510(k) Summary for the Somatom Definition AS Open. https://www.accessdata.fda.gov/scripts/cdrh/cfdocs/cfpmn/pmn.cfm?ID=K130901; 2014.

[12] Philips Medical Systems (Cleveland), Inc . 510(k) Summary for Philips Ingenuity CT. https://www.accessdata.fda.gov/scripts/cdrh/cfdocs/cfpmn/pmn.cfm?ID=K160743; 2016.

[13] GE Medical Systems, L.L.C. 510(k) Summary for Revolution CT. https://www.accessdata.fda.gov/scripts/cdrh/cfdocs/cfpmn/pmn.cfm?ID=K163213; 2016.

[14] Glover GH , Pelc NJ. An algorithm for the reduction of metal clip artifacts in CT reconstructions. Med Phys. 1981;8:799–807. 7322078 10.1118/1.595032

[15] Kalender W , Hebel R , Ebersberger J. Reduction of CT artifacts caused by metallic implants. Radiology. 1987;164:576–577. 3602406 10.1148/radiology.164.2.3602406

[16] Karimi S , Cosman P , Wald C , Martz H. Segmentation of artifacts and anatomy in CT metal artifact reduction. Med Phys. 2012;39:5857–5868. 23039624 10.1118/1.4749931

[17] Veldkamp WJ , Joemai R , van der Molen AJ , Geleijns J. Development and validation of segmentation and interpolation techniques in sinograms for metal artifact suppression in CT. Med Phys. 2010;37:620–628. 20229871 10.1118/1.3276777

[18] Hinderling T , Rùegsegger P , Anliker M , Dietschi C . Computed tomography reconstruction from hollow projections: an application to in vivo evaluation of artificial hip joints. J Comput Assist Tomogr. 1979;3:52–57. 422792

[19] Klotz E , Kalender WA , Sokiransky R , Felsenberg D . Algorithms for the reduction of CT artifacts caused by metallic implants In: Medical Imaging IV: PACS Systems Design and Evaluation, volume 1234, Bellingham, WA: International Society for Optics and Photonics; 1990:642–651.

[20] Mahnken AH , Raupach R , Wildberger JE , et al. A new algorithm for metal artifact reduction in computed tomography: in vitro and in vivo evaluation after total hip replacement. Invest Radiol. 2003;38:769–775. 14627894 10.1097/01.rli.0000086495.96457.54

[21] Zhao S , Robeltson D , Wang G , Whiting B , Bae KT. X‐ray CT metal artifact reduction using wavelets: an application for imaging total hip prostheses. IEEE Trans Med Imaging. 2000;19:1238–1247. 11212372 10.1109/42.897816

[22] Prell D , Kyriakou Y , Struffert T , D“orfler A , Kalender W. Metal artifact reduction for clipping and coiling in interventional C‐arm CT. Am J Neuroradiol. 2010;31:634–639. 19942707 10.3174/ajnr.A1883 PMC7964233

[23] Meyer E , Raupach R , Lell M , Schmidt B , Kachelriess M. Normalized metal artifact reduction (NMAR) in computed tomography. Med Phys. 2010;37:5482–5493. 21089784 10.1118/1.3484090

[24] Gjesteby L , De Man B , Jin Y , et al. Metal artifact reduction in CT: where are we after four decades? IEEE Access. 2016;4:5826–5849.

[25] Desai SD , Kulkarni L. Comprehensive survey on metal artifact reduction methods in computed tomography images. Int J Rough Sets Data Anal. 2015;2:92–114.

[26] Lemmens C , Faul D , Nuyts J. Suppression of metal artifacts in CT using a reconstruction procedure that combines MAP and projection completion. IEEE Trans Med Imaging. 2009;28:250–260. 19188112 10.1109/TMI.2008.929103

[27] Zhang X , Wang J , Xing L. Metal artifact reduction in x‐ray computed tomography (CT) by constrained optimization. Med Phys. 2011;38:701–711. 21452707 10.1118/1.3533711 PMC3033877

[28] Morsbach F , Bickelhaupt S , Wanner GA , Krauss A , Schmidt B , Alkadhi H. Reduction of metal artifacts from hip prostheses on CT images of the pelvis: value of iterative reconstructions. Radiology. 2013;268:237–244. 23513244 10.1148/radiol.13122089

[29] Chen Y , Li Y , Guo H , et al. CT metal artifact reduction method based on improved image segmentation and sinogram in‐painting. Math Probl Eng. 2012;2012:1–18.

[30] Guggenberger R , Winklhofer S , Osterhoff G , et al. Metallic artefact reduction with monoenergetic dual‐energy CT: systematic ex vivo evaluation of posterior spinal fusion implants from various vendors and different spine levels. Eur Radiol. 2012;22:2357–2364. 22645043 10.1007/s00330-012-2501-7

[31] Coupal TM , Mallinson PI , McLaughlin P , Nicolaou S , Munk PL , Ouellette H. Peering through the glare: using dual‐energy CT to overcome the problem of metal artefacts in bone radiology. Skeletal Radiol. 2014;43:567–575. 24435711 10.1007/s00256-013-1802-5

[32] Filograna L , Magarelli N , Leone A , et al. Value of monoenergetic dual‐energy CT (DECT) for artefact reduction from metallic orthopedic implants in post‐mortem studies. Skeletal Radiol. 2015;44:1287–1294. 25962510 10.1007/s00256-015-2155-z

[33] Han SC , Chung YE , Lee YH , Park KK , Kim MJ , Kim KW. Metal artifact reduction software used with abdominopelvic dual‐energy CT of patients with metal hip prostheses: assessment of image quality and clinical feasibility. Am J Roentgenol. 2014;203:788–795. 25247944 10.2214/AJR.13.10980

[34] Kuchenbecker S , Faby S , Sawall S , Lell M , Kachelriess M. Dual energy CT: how well can pseudo‐monochromatic imaging reduce metal artifacts? Med Phys. 2015;42:1023–1036. 25652515 10.1118/1.4905106

[35] De Man B . Method and apparatus for the reduction of artifacts in computed tomography images. http://www.patentlens.net/patentlens/patent/US_7444010/en/; 2008.

[36] Hitachi Medical Systems America Inc . 510(k) Summary for the HITACHI Supria Whole‐body X‐ray CT System Phase 3; http://www.accessdata.fda.gov/cdrh_docs/pdf16/K163528.pdf; 2016.

[37] Boas FE , Fleischmann D. Evaluation of two iterative techniques for reducing metal artifacts in computed tomography. Radiology. 2011;259:894–902. 21357521 10.1148/radiol.11101782

[38] Li H , Noel C , Chen H , et al. Clinical evaluation of a commercial orthopedic metal artifact reduction tool for CT simulations in radiation therapy. Med Phys. 2012;39:7507–7517. 23231300 10.1118/1.4762814 PMC3618095

[39] Google search on character string “ CT compatible . Accessed: 2018‐02‐22.

[40] Kolind SH , MacKay AL , Munk PL , Xiang Q‐S. Quantitative evaluation of metal artifact reduction techniques. J Magn Reson Imaging. 2004;20:487–495. 15332257 10.1002/jmri.20144

[41] Bal M , Spies L. Metal artifact reduction in CT using tissue‐class modeling and adaptive prefiltering. Med Phys. 2006;33:2852. 16964861 10.1118/1.2218062

[42] De Man B , Nuyts J , Dupont P , Marchal G , Suetens P. An iterative maximum‐likelihood polychromatic algorithm for CT. IEEE Trans Med Imaging. 2001;20:999–1008. 11686446 10.1109/42.959297

[43] Silverstein DA , Farrell JE . The relationship between image fidelity and image quality. In: *Proceedings, International Conference on Image Processing*, volume 1, IEEE; 1996:881–884.

[44] Wang Z , Bovik AC , Lu L . Why is image quality assessment so difficult? In: *Acoustics, Speech, and Signal Processing (ICASSP)*, 2002 IEEE International Conference on, volume 4, pages IV‐3313. IEEE; 2002.

[45] Bolstad K , Flatabo S , Aadnevik D , Dalehaug I , Vetti N. Metal artifact reduction in CT, a phantom study: subjective and objective evaluation of four commercial metal artifact reduction algorithms when used on three different orthopedic metal implants. Acta Radiol. 2018;59:1110–1118. 29310445 10.1177/0284185117751278

[46] Hu Y , Pan S , Zhao X , Guo W , He M , Guo Q. Value and clinical application of orthopedic metal artifact reduction algorithm in CT scans after orthopedic metal implantation. Kor J Radiol. 2017;18:526–535. 10.3348/kjr.2017.18.3.526 PMC5390622 28458605

[47] Subhas N , Polster JM , Obuchowski NA , et al. Imaging of arthroplasties: improved image quality and lesion detection with iterative metal artifact reduction, a new CT metal artifact reduction technique. Am J Roentgenol. 2016;207:378–385. 27186794 10.2214/AJR.15.15850

[48] Barrett HH , Myers KJ . Foundations of Image Science. New York: Wiley; 2004.

[49] Wani AA , Ramzan AU , Shoib Y , et al. Stray bullet: an accidental killer during riot control. Surg Neurol Int. 2011;2:122. 22022659 10.4103/2152-7806.84769 PMC3198307

[50] Hanpeter DE , Demetriades D , Asensio JA , Berne TV , Velmahos G , Murray J. Helical computed tomographic scan in the evaluation of mediastinal gunshot wounds. J Trauma Acute Care Surg. 2000;49:689–695. 10.1097/00005373-200010000-00017 11038087

[51] Hesterman JY , Kupinski MA , Clarkson E , Barrett HH. Hardware assessment using the multi‐module, multi‐resolution system (M3R): a signal‐detection study. Med Phys. 2007;34:3034–3044. 17822011 10.1118/1.2745920 PMC2471875

[52] Alvarez RE . A Matlab function to compute the attenuation coefficient, http://www.aprendtech.com/blog/Post2/xraymu.html; 2011.

[53] Berger M , Seltzer S . XCOM Photon Cross Sections, volume 3; 1999.

[54] LaRiviére PJ. Penalized‐likelihood sinogram smoothing for low‐dose CT. Med Phys. 2005;32:1676–1683. 16013726 10.1118/1.1915015

[55] Mehranian A , Ay MR , Rahmim A , Zaidi H. 3D prior image constrained projection completion for X‐ray CT metal artifact reduction. IEEE Trans Nucl Sci. 2013;60:3318–3332.

[56] Siemens Healthineers . Online tool for the simulation of X‐ray Spectra. https://www.oem‐products.siemens.com/x‐ray‐spectra‐simulation; 2016.

[57] MATLAB . Release. Natick, MA, USA: The MathWorks, Inc.; 2017b.

[58] Vaishnav J , Jung W , Popescu L , Zeng R , Myers K. Objective assessment of image quality and dose reduction in CT iterative reconstruction. Med Phys. 2014;41:071904. 24989382 10.1118/1.4881148

[59] Abbey CK , Barrett HH. Human and model‐observer performance in ramp‐spectrum noise: effects of regularization and object variability. J Opt Soc Am A. 2001;18:473–488. 10.1364/josaa.18.000473 PMC2943344 11265678

[60] Wunderlich A , Noo F , Gallas BD , Heilbrun ME. Exact confidence intervals for channelized Hotelling observer performance in image quality studies. IEEE Trans Med Imaging. 2015;34:453–464. 25265629 10.1109/TMI.2014.2360496 PMC5542023

[61] Wagner R , Yousef W , Chen W . Finite training of radiologists and statistical learning machines: parallel lessons In:Wolbarst AB, Hendee WR, Mossman KL, eds. Advances in Medical Physics 2008. Chapter 9. Madison, WI: Medical Physics Publishing; 2008:129–141.

[62] Myers KJ , Chen W. Special Section guest editorial: pioneers in medical imaging: honoring the memory of Robert F. Wagner. J Med Imaging. 2014;1:031001. 10.1117/1.JMI.1.3.031001 PMC4489347 26158043

[63] Chan H‐P , Sahiner B , Wagner RF , Petrick N. Classifier design for computer‐aided diagnosis: effects of finite sample size on the mean performance of classical and neural network classifiers. Med Phys. 1999;26:2654–2668. 10619251 10.1118/1.598805

[64] Fukunaga K. Introduction to Statistical Pattern Recognition. Cambridge, MA: Academic Press; 2013.

[65] iMRMC software . https://github.com/DIDSR/iMRMC.

[66] Popescu LM , Myers KJ. CT image assessment by low contrast signal detectability evaluation with unknown signal location. Med Phys. 2013;40:111908. 24320441 10.1118/1.4824055

[67] Gottlieb S . How FDA Plans to Help Consumers Capitalize on Advances in Science. https://www.fda.gov/NewsEvents/Newsroom/FDAVoices/ucm612016.htm; 2016. Accessed: 2018‐02‐22.

[68] Faris O , Shuren J. An FDA viewpoint on unique considerations for medical‐device clinical trials. N Engl J Med. 2017;376:1350–1357. 28379790 10.1056/NEJMra1512592

[69] Badano A , Graff CG , Badal A , et al. Evaluation of digital breast tomosynthesis as replacement of full‐field digital mammography using an in silico imaging trial: digital breast tomosynthesis as replacement of full‐field digital mammography. JAMA Netw Open. 2018;1:e185474–e185474. 30646401 10.1001/jamanetworkopen.2018.5474 PMC6324392

